# Fish bone migration into the trachea: gastroscopic removal and esophagotracheal fistula closure

**DOI:** 10.1055/a-2991-2332

**Published:** 2026-11-03

**Authors:** Xi Wang, Rui Zhong, Xiangsheng Fu, Kui Zhao

**Affiliations:** 1 Department of Gastroenterology 580504 Clinical Medical College and The First Affiliated Hospital of Chengdu Medical CollegeChengduChina

A 53-year-old man presented with a 3-day history of foreign body sensation and pharyngeal pain after fish consumption, followed by progressive cough, sputum production, and mild dyspnea. Computed tomography (CT) demonstrated a high-density linear structure resembling a fishbone lodged in the upper esophagus, penetrating through the esophageal wall and partially extending into the tracheal lumen ( Fig. 1 ). Endoscopy was performed under intravenous general anesthesia. Esophagoscopy revealed localized swelling and ulceration of the anterior esophageal wall with a fistulous opening and purulent drainage, but no foreign body was found in the esophageal lumen ( Fig. 2 ), suggesting migration beyond the esophageal wall. Given the CT findings suggesting airway penetration, the endoscope was carefully advanced through the glottis into the trachea, where the migrated fishbone was identified ( Fig. 3 ). The fishbone was carefully grasped and retrieved through the tracheal route using foreign body forceps ( Fig. 4 ). After removal, the extracted fishbone was measured to be approximately 2.5 cm in length ( Fig. 5 ). Repeat esophagoscopy revealed the esophageal perforation defect at the previous penetration site. After thorough irrigation, the defect was successfully closed from the esophageal side using three metal clips to prevent persistent esophagotracheal communication ( Video 1 ). A liquid diet was initiated 24 hours after the procedure. Due to initial purulent drainage and respiratory symptoms, antibiotic therapy was continued. The patient was discharged 3 days later with significant symptom improvement. During 1-month follow-up, he remained asymptomatic without dysphagia or respiratory complications. This case highlights a rare complication of esophageal foreign body ingestion, in which a fishbone penetrated the esophageal wall and migrated into the trachea. Careful interpretation of imaging findings and evaluation of adjacent structures are essential when the foreign body is not visualized during esophagoscopy. In selected cases, endoscopic retrieval through the airway and closure of the esophageal defect may provide a minimally invasive alternative to surgical management.

**Fig. 1:**
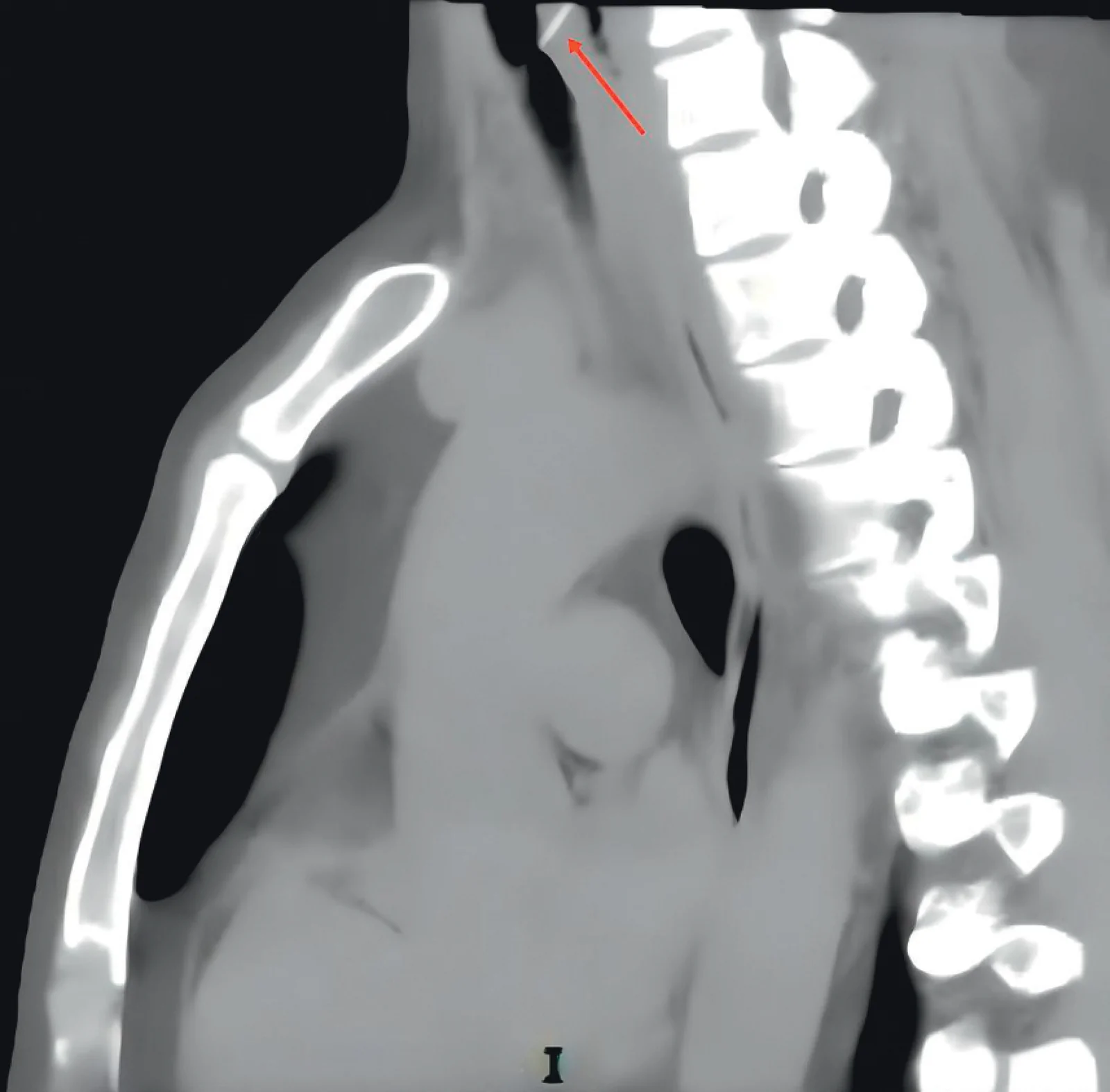
A sagittal computed tomographic (CT) image showing a linear hyperdense foreign body (arrow) located in the upper esophagus, penetrating the esophageal wall and extending into the adjacent tracheal lumen.

**Fig. 2:**
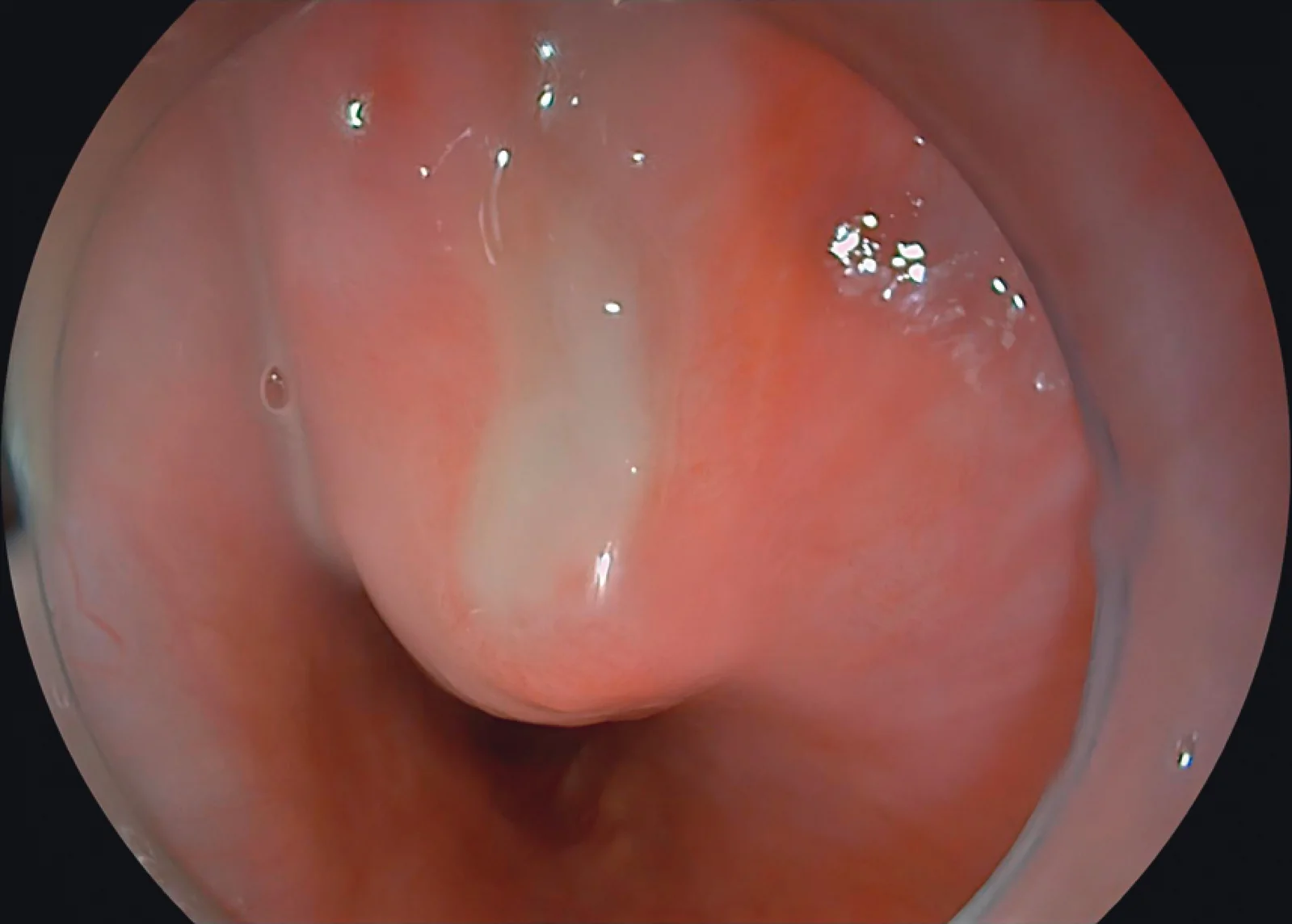
An endoscopic view of the fistulous opening with purulent discharge at the upper esophageal inlet.

**Fig. 3:**
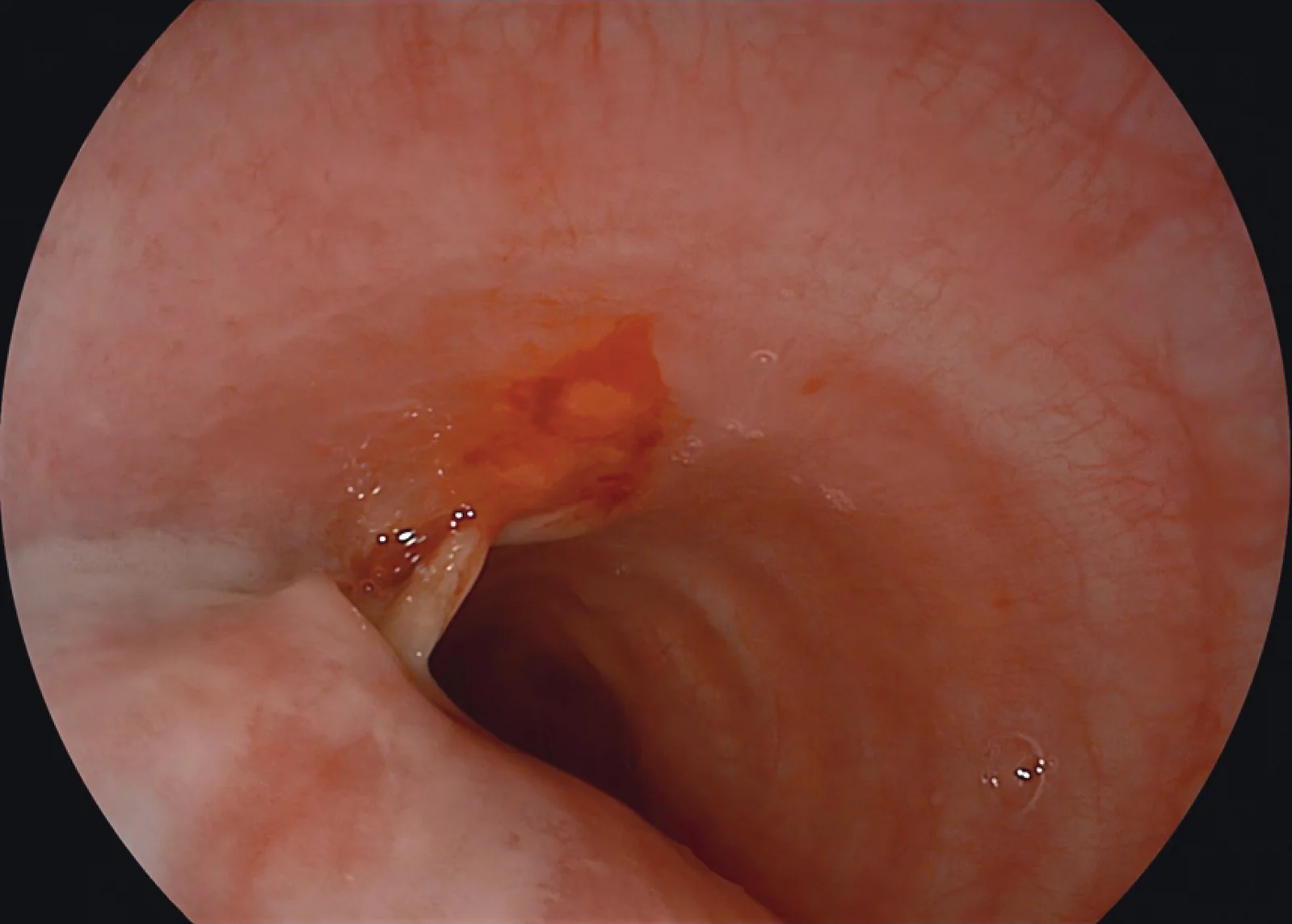
An endoscopic view of the fishbone protruding into the tracheal lumen.

**Fig. 4:**
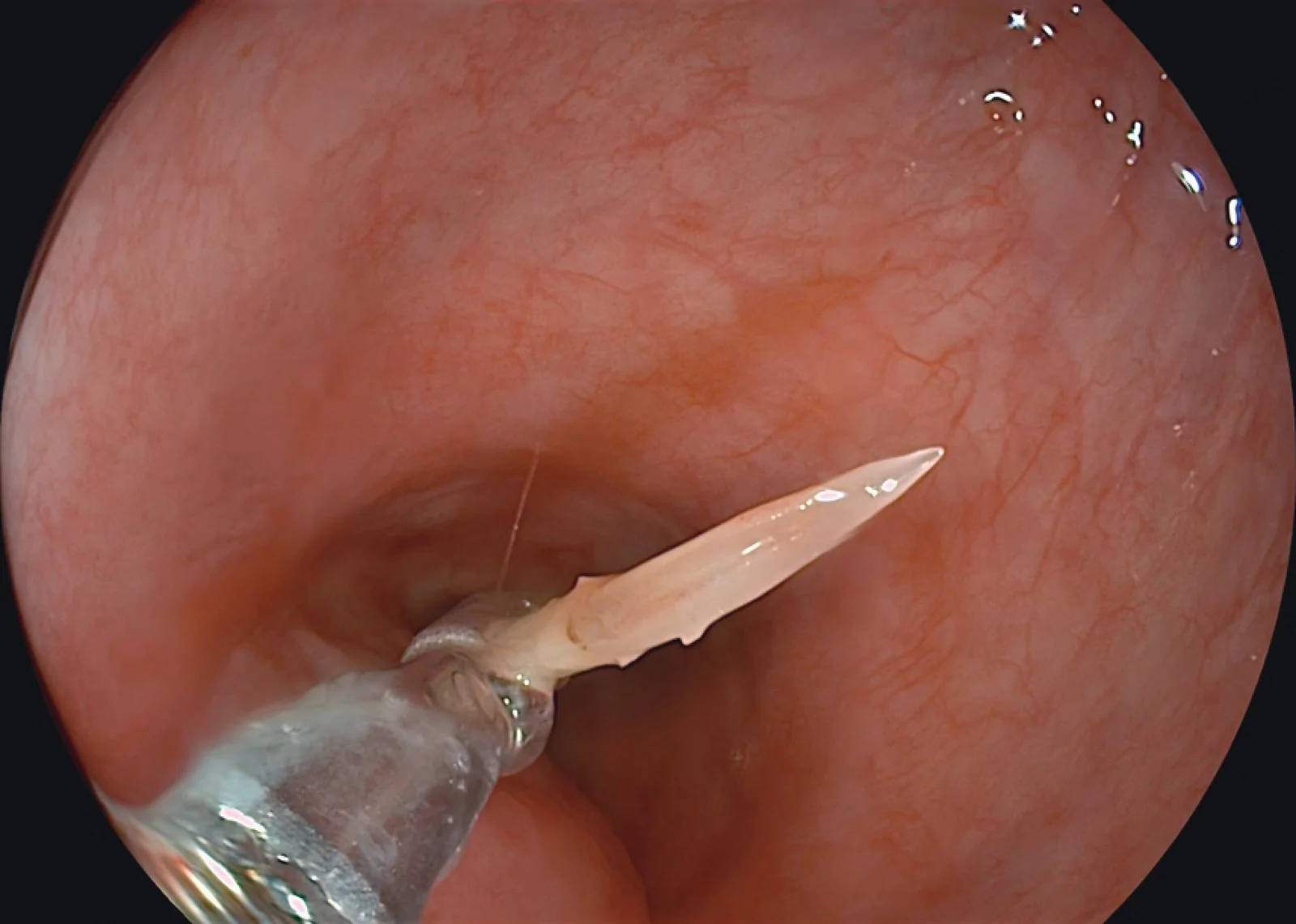
Endoscopic retrieval of the fishbone using foreign body forceps through the tracheal route.

**Fig. 5:**
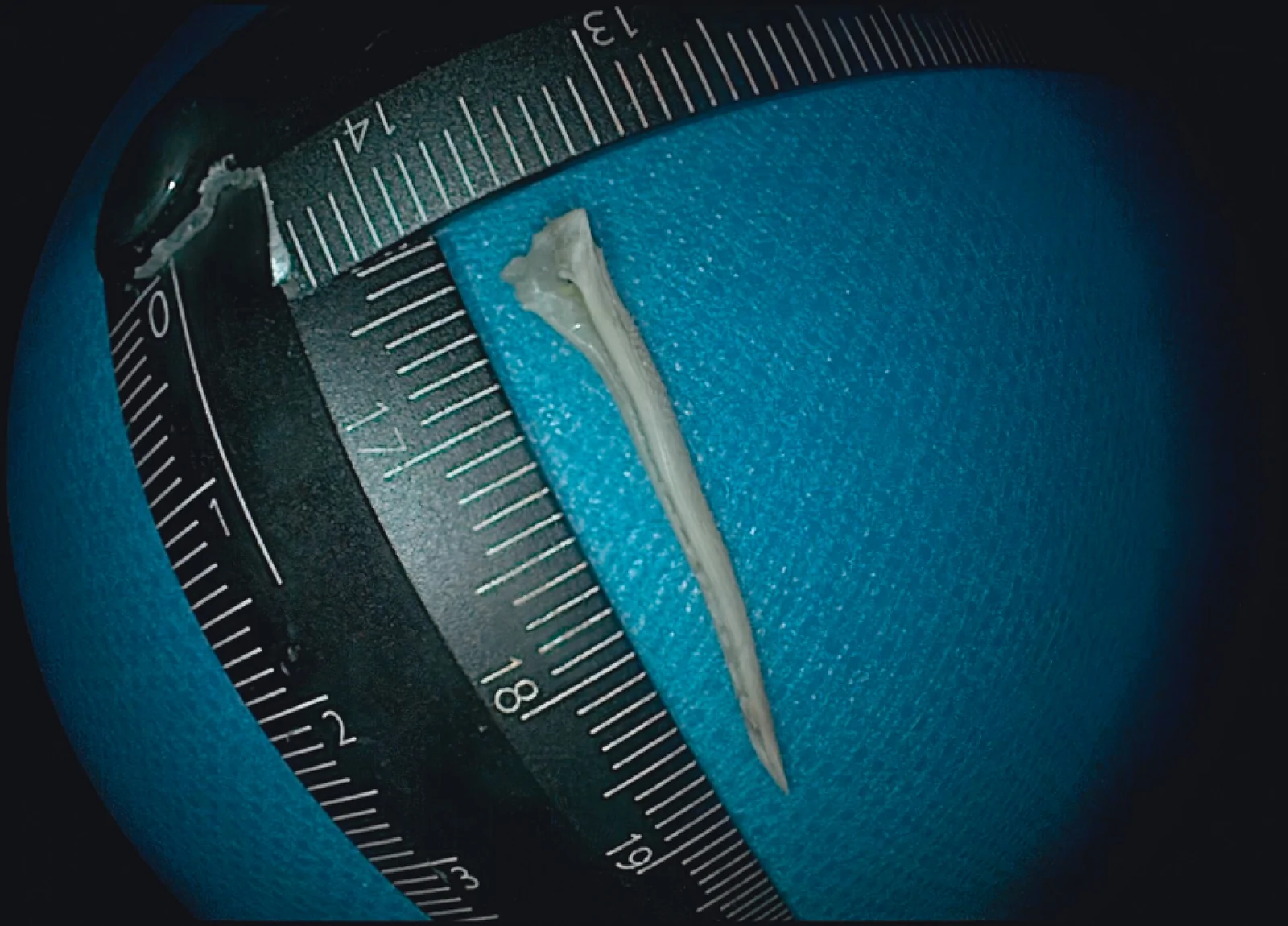
The extracted fishbone measuring approximately 2.5 cm in length.

**Video 1** Endoscopic clip closure of the esophageal perforation after tracheal retrieval of the penetrating fishbone. URI: .


Endoscopy_UCTN_Code_TTT_1AO_2AL g

## Declaration of GenAI Use

The authors confirm that no generative artificial intelligence was used in the preparation of this manuscript.

